# RNA-Seq Transcriptomics and iTRAQ Proteomics Analysis Reveal the Dwarfing Mechanism of Blue Fescue (*Festuca glauca*)

**DOI:** 10.3390/plants13233357

**Published:** 2024-11-29

**Authors:** Yong Zhang, Peng Han, Ruijie Zhao, Shuhan Yu, Hang Liu, Hong Wu, Jinyang Weng, Hengfeng Zhang

**Affiliations:** 1 School of Landscape Architecture and Horticulture, Jiangsu Agri-Animal Husbandry Vocational College, Taizhou 225300, China; 2022010535@jsahvc.edu.cn (Y.Z.); liuhang316022@126.com (H.L.); wh02gs@126.com (H.W.); wengjinyang@jsahvc.edu.cn (J.W.); 2 College of Agro-Grassland Science, Nanjing Agricultural University, Nanjing 210095, China; 19852869215@163.com (P.H.); zrj152342767970520@163.com (R.Z.); 3 College of Landscape Architecture, Zhejiang A&F University, Hangzhou 311300, China; 20220144@zafu.edu.cn

**Keywords:** blue fescue, dwarfing mechanism, GAs, IAA, lignin

## Abstract

Blue fescue is a widely used ornamental grass because of its strong ecological adaptability. To maintain the optimal ornamental plant shape, blue fescue requires many nutrients and labor. Using dwarf varieties with slow growth is an effective way to fulfill these requirements. In this study, we investigated the dwarfing mechanism of *dw-1*, a blue fescue dwarfing mutant, using physiological, transcriptomic, and proteomic methods. The peroxidase (POD) enzyme activity and chlorophyll content of *dw-1* significantly increased, while the lignin, gibberellin (GA), and indoleacetic acid (IAA) content significantly decreased. A total of 7668 differentially expressed genes (DEGs) were detected using RNA-seq, of which 2543 were upregulated and 5125 were downregulated. A total of 165 differentially expressed proteins (DEPs) were detected using iTRAQ, of which 68 were upregulated and 97 were downregulated. KEGG enrichment analysis showed that the diterpene biosynthesis pathway, tryptophan metabolism pathway, and phenylpropanoid biosynthesis pathway were significantly enriched at both the transcriptional and protein levels. As a result, we can formulate the following hypothesis about the *dw-1* dwarfing phenotype: the downregulation of genes and proteins related to IAA and GA biosynthesis is associated with the dwarf phenotype’s formation, and metabolic pathways related to lignin synthesis, such as phenylpropanoid biosynthesis, also play an important role. Our work will contribute to a new understanding of the genes and proteins involved in the blue fescue dwarf phenotype.

## 1. Introduction

Blue fescue (*Festuca glauca*) is a perennial cold-season ornamental grass [1]. It has many advantages, such as strong stress resistance, simple maintenance and management, and high ornamental value. Therefore, it is mainly used for flower beds, flower borders, or road edges in garden greening, and it has significant market development potential [2]. Blue fescue has a taller cluster and is not suitable for turfgrass. To maintain the optimal plant shape for blue fescue, it is necessary to regularly carry out maintenance work such as pruning, fertilization, and irrigation, which requires a lot of labor and material resources. Dwarf varieties have a slower growth rate and lower nutrient consumption, which is important for reducing labor intensity, reducing water consumption, and improving fertilizer utilization efficiency [3]. Meanwhile, cultivating turf-type blue fescue could expand its applications in the sports field. Therefore, it is necessary to cultivate new varieties of low-plant-type blue fescue.

Dwarfing is an important agronomic trait in crops and turfgrass, and it has become an important research direction for breeding excellent crop varieties over long periods [4,5,6]. There are many reasons for plant dwarfing phenotypes; for example, hormone metabolism and signal transduction affecting cell division or elongation are significant causes of plant dwarfing [7,8,9,10]. Studies have shown that plant hormones such as brassinosteroids (BRs), gibberellins (GAs), and indoleacetic acid (IAA) are mainly involved in forming dwarf mutants [11]. Plant-hormone-related dwarf mutants can be divided into hormone-synthesis-deficient and hormone-insensitive mutants [12,13]. Among them, GA-related dwarf varieties played an important role in the green revolution in the 1960s [14]. The “green revolution” gene *sd1* (*semi-dwarf 1*) in rice (*Oryza sativa*) encodes a key enzyme, GA20ox2, in the GA biosynthesis pathway; the “green revolution” gene *Rht1* (*Reduced height 1*) in wheat (*Triticum aestivum*) encodes a key regulatory element, the DELLA protein, in the GA signal transduction pathway [15,16,17,18]. Meanwhile, mutants lacking polyamines, salicylic acid (SA), and strigolactone (SL) in rice and *Arabidopsis thaliana* also show dwarf phenotypes [19,20,21,22].

In addition, the mutation of some genes in the phenylpropanoid biosynthesis pathway can also lead to dwarf phenotypes. Lignin is one of the metabolites of the phenylpropanoid pathway [23], and silencing or knocking out some key lignin monomer biosynthesis genes will lead to a decline in plant height, accompanied by reduced lignin content [24]. The defective expression of *shikimate/quinic acid hydroxycinnamoyl transferase* (*HCT*) or *p-coumaroyl ester 3′-hydroxylase* (*C3′H*) leads to defective phenylpropionic acid biosynthesis in *Arabidopsis thaliana*, resulting in reduced lignin content, flavonoid enrichment, and growth inhibition, indicating that plant lignification plays a crucial role in regulating plant height [25].

Transcriptomics and proteomics have wide applications in exploring the plant dwarfism mechanism. A proteomic study of the dwarf mutant *LA-1* in upland cotton revealed that the DELLA-independent GA signaling pathway is the primary cause of dwarfism in *LA-1* [26]. A transcriptome and proteome analysis of the dwarf rapeseed variety *DW871* revealed that plant hormone signaling, such as IAA and BRs, is associated with dwarf phenotype formation, while metabolic pathways related to lignin synthesis, such as phenylpropanoid biosynthesis, also play a significant role [27]. A transcriptome analysis of the *Zoysia matrella* dwarf mutant revealed that downregulating differentially expressed genes (DEGs) related to IAA transport and cell wall development may cause dwarfism in *Zoysia matrella* [28]. An analysis of the transcriptome and proteome of the T51 dwarf mutant of seashore paspalum (*Paspalum vaginatum*) revealed that its dwarf phenotype is closely associated with the downregulation of lignin-synthesis-related genes and a decrease in lignin content [29]. However, there is no relevant report on the dwarfing mechanism of blue fescue. Presently, the research on blue fescue mainly focuses on stress resistance [1], with none on its genes and genomics. The lack of genomic information poses an obstacle to developing new varieties of blue fescue through modern genetic and genomic methods. For non-model species that lack a sequenced genome, such as blue fescue, RNA-Seq is a valuable tool for developing new genetic resources. In this study, we combined RNA-Seq and iTRAQ to construct unique transcripts and proteomes of *dw-1* and the WT. We then screened dwarfism-related genes and proteins to understand the dwarfing mechanism of *dw-1*. This study provides transcriptome and proteome profiles for blue fescue dwarfing mutants and supports a foundation for future gene screening related to dwarfing in this plant.

## 2. Results

### 2.1. Phenotypic Characterization of dw-1 and the WT

**Figure 1:**
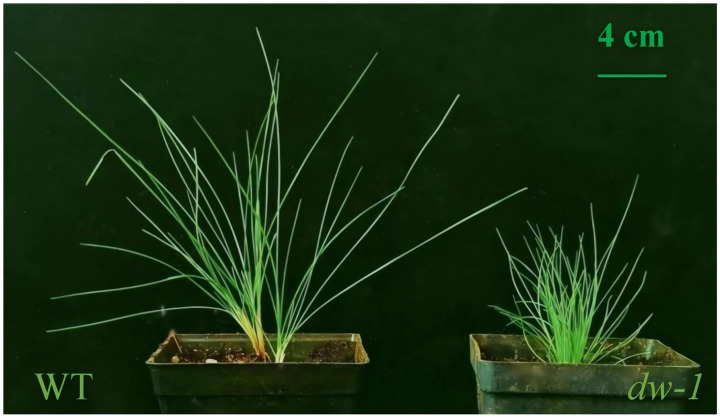
Phenotypic characteristics of the WT and *dw-1*.

Unlike the WT, *dw-1* showed a dwarf phenotype (Figure 1). The plant height and leaf length of *dw-1* were significantly shorter than those of the WT (Table 1). There was no significant change in the tiller number between *dw-1* and the WT. Additionally, the aboveground biomass of *dw-1* was significantly lower than that of the WT, and there was no significant difference in the underground biomass (Table 1).

### 2.2. Identification of DEGs

**Figure 2:**
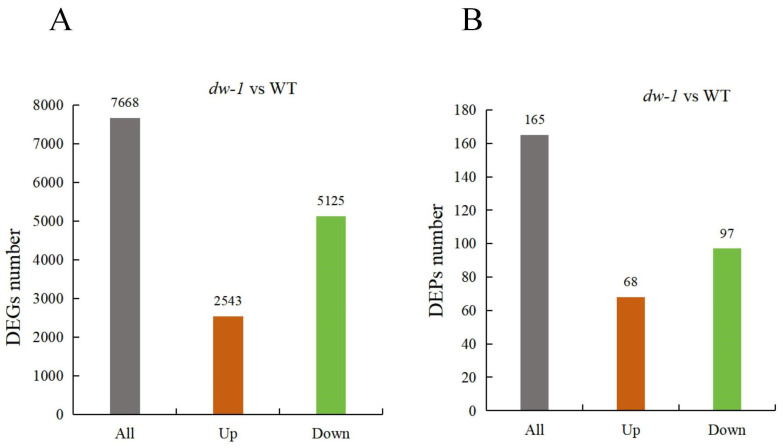
Statistics for DEGs and DEPs between *dw-1* and the WT. (**A**) DEG number between *dw-1* and the WT; (**B**) DEP number between *dw-1* and the WT.

To verify the difference in transcript levels between the mutant *dw-1* and the WT, we constructed six cDNA libraries (i.e., WT-1, WT-2, WT-3, dw-1, dw-2, and dw-3) using total RNA extracted from the WT and *dw-1* leaves. After the library was qualified, the high-throughput sequencer MGI was used for sequencing to obtain raw reads (accession number: PRJNA1166912). Subsequently, the quality of the sequencing data was evaluated, and the statistical items included the number of clean reads, the number of clean bases, the value of Clean GC, the value of Clean Q20, and the value of Clean Q30 (Table S1). Trinity software (v2.4.0) was used to assemble transcripts (Table S2), and bowtie2 software (v2.1.0) was used to compare the quality-controlled sequences of each sample with the reference transcript sequences (Table S3). The transcripts were annotated with basic functions in the NR, GO, KOG, KEGG, and Swiss-Prot databases (Table S4). Finally, compared with the WT, we obtained 7668 DEGs in *dw-1*, of which 2543 were upregulated and 5125 were downregulated (Figure 2A).

### 2.3. Identification of DEPs

To clarify the dwarfing phenotype formation mechanism, we detected the differentially expressed proteins (DEPs) between *dw-1* and the WT using iTRAQ proteomics. Protein sequencing from *dw-1* and WT samples yielded 396,766 spectra, 22,712 of which were unique peptides (Table S5). They could identify 3520 proteins (Table S5). The total proteins were annotated with basic functions in the GO, KOG, and KEGG databases (Table S6). Finally, compared with the WT, we obtained 165 DEPs in *dw-1*, of which 68 were upregulated and 97 were downregulated (Figure 2B).

### 2.4. KEGG Enrichment Analysis of DEGs

**Figure 3:**
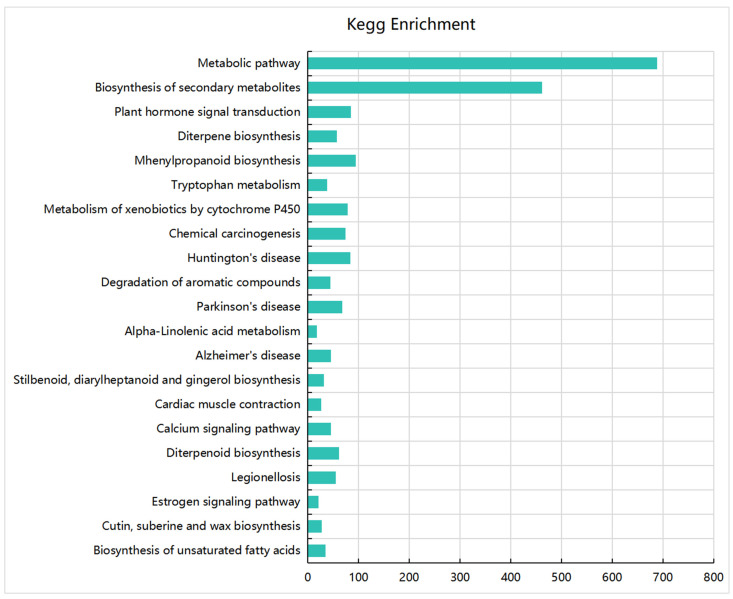
KEGG pathway enrichment analysis of DEGs. The y-axis represents metabolic pathways, and the x-axis represents the number of DEGs enriched in the metabolic pathway.

To verify which metabolic pathways the DEGs are involved in and screen dwarf-related genes, we performed a KEGG enrichment analysis. The results showed that the DEGs were significantly enriched in 21 metabolic pathways, of which the most enriched pathways were the metabolic pathway and the biosynthesis of secondary metabolites (Figure 3). In addition, the DEGs were significantly enriched in the plant hormone signal transduction, diterpene biosynthesis, phenylamine metabolism, and tryptophan metabolism pathways, and these metabolic pathways were closely related to the dwarf phenotype (Figure 3).

### 2.5. KEGG Enrichment Analysis of DEPs

**Figure 4:**
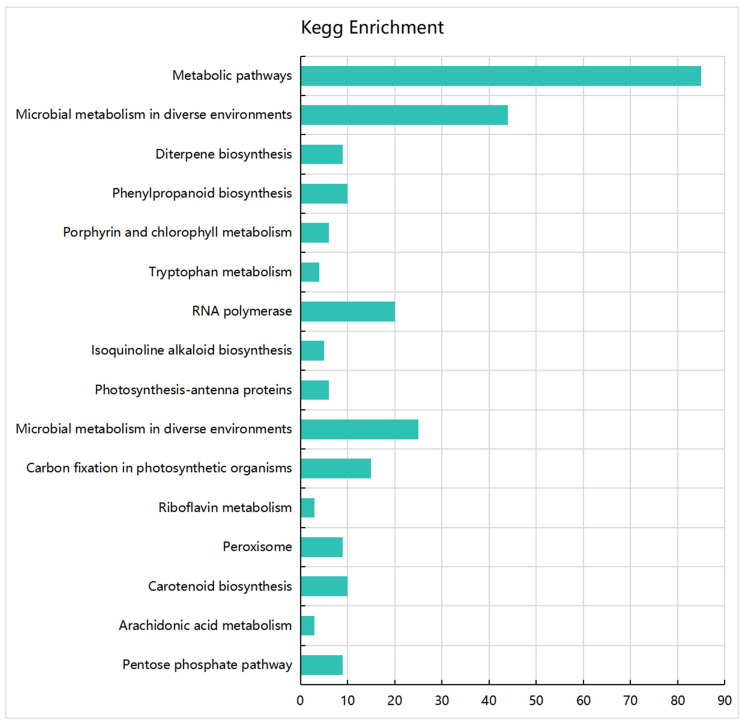
KEGG pathway enrichment analysis of DEPs. The y-axis represents metabolic pathways, and the x-axis represents the number of DEPs enriched in the metabolic pathway.

To verify the expression of the DEPs at the protein level, we performed iTRAQ proteome analysis on *dw-1* and the WT and then performed KEGG pathway enrichment analysis on the DEPs obtained between *dw-1* and the WT. The DEPs were significantly enriched in 16 metabolic pathways, of which the most enriched were the metabolic pathway and microbial metabolism in diverse environments (Figure 4). In addition, the DEPs were also significantly enriched in the diterpene biosynthesis, phenylamine metabolism, and tryptophan metabolism pathways, and these metabolic pathways were closely related to the dwarf phenotype (Figure 4).

### 2.6. Selection of Key Genes Related to Dwarfism

In organisms, mRNA and proteins correspond to different stages of gene expression. mRNA is only an intermediate process in gene expression, whereas proteins are the final products of this process. Therefore, proteomics can more accurately represent the phenotypes of plants than transcriptomics. For this study, we screened genes that were significantly differentially expressed at both the transcriptional and protein levels as key dwarf genes in the *dw-1* mutant. In the diterpene biosynthesis pathway, 58 DEGs and 9 DEPs were significantly enriched, and their annotation positions in the KEGG pathway are shown in Figure S1 and Figure S2, respectively. Nine DEPs were involved in GA biosynthesis, among which five DEPs, including one CPS, one KO, and three GA2oxes, were upregulated, and four DEPs, including one KS, two GA20oxes, and one GA3ox, were downregulated (Table 2). Of these, the *GA20ox-2*, *GA2ox-1*, and *GA2ox-2* genes exhibited the greatest changes at the transcription and protein levels and can be considered candidate genes responsible for the dwarf phenotype of *dw-1* (Table 2).

In the tryptophan metabolism pathway, 38 DEGs and 4 DEPs were significantly enriched, and their annotation positions in the KEGG pathway are shown in Figure S3 and Figure S4, respectively. Four DEPs were involved in IAA biosynthesis, of which two TAA1 and two YUCCAs were downregulated (Table 2). Of these, the *TAA1-2* and *YUCCA-1* genes exhibited the greatest changes at the transcription and protein levels and can be considered candidate genes responsible for the dwarf phenotype of *dw-1* (Table 3).

In the phenylpropanoid biosynthesis pathway, 95 DEGs and 10 DEPs were significantly enriched, and their annotation positions in the KEGG pathway are shown in Figure S5 and Figure S6, respectively. Ten DEPs were involved in lignin biosynthesis, of which five, including one PAL, one 4CL, and three peroxidases were upregulated, and five DEPs, including two CCRs, one HCT, one CAD, and one COMT, were downregulated (Table 4). Of these, the *CCR-1* and *CAD* genes exhibited the greatest changes at the transcription and protein levels and can be considered candidate genes responsible for the dwarf phenotype of *dw-1*.

### 2.7. Validation of RNA-Seq Results

**Figure 5:**
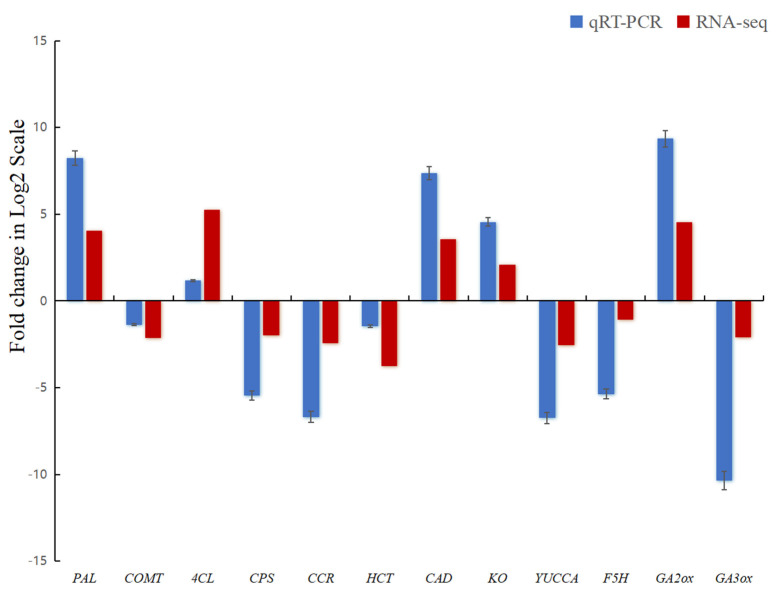
qRT-PCR verification of DEGs between the WT and *dw-1*. Ordinate shows the logarithm of the differential multiples of the corresponding gene, and the positive and negative values of the y-axis express the gene up and down, respectively.

To verify the reliability of our transcriptome sequencing results, we detected and analyzed 12 DEGs in the GA, IAA, and phenylpropanoid biosynthesis pathways using qRT-PCR, of which 5 were upregulated and 7 were downregulated. The gene expression changes found with qRT-PCR are consistent with the RNA-seq analysis results, although the fold change in gene differential expression may differ between the two technologies (Figure 5). These results indicate that the RNA-seq data in the present study are reliable.

### 2.8. Changes in Physiological Levels

Physiological and biochemical changes in plants are often used to measure variation. In this study, we measured the activities of antioxidant enzymes (CAT, POD and SOD), chlorophyll content, lignin content, cellulose content, H_2_O_2_ content, and O_2_^−^ content in the fresh mature leaves of *dw-1* and the WT. POD activity significantly increased in *dw-1*, and CAT and SOD activities were slightly higher without significant changes (Table 5). The chlorophyll a and b contents of *dw-1* significantly increased, and the Chla/Chlb ratios in *dw-1* and the WT were 2.12 and 2.49, respectively (Table 5 and Table S7). The increase in chlorophyll content is consistent with the greener phenotype of *dw-1* leaves. Compared with the WT, the lignin and cellulose content in the *dw-1* leaves decreased significantly (Table 4). However, there was no significant difference in the H_2_O_2_ and O_2_^−^ content between *dw-1* and the WT. These changes clarify the differences between *dw-1* and the WT at the physiological level.

### 2.9. Changes in Endogenous Hormone Content

**Figure 6:**
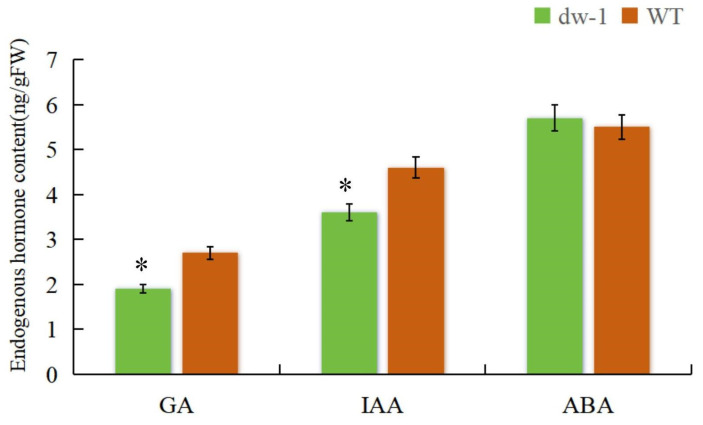
Endogenous hormone content of *dw-1* and the WT. Note: * indicates a significant difference in Duncan’s multiple comparisons, where *p* ≤ 0.05, *n* = 6.

To better understand the role of plant hormones in the *dw-1* dwarf mutant, the contents of three major endogenous hormones in *dw-1* and WT leaves were determined. Compared with the WT, the contents of ABA in *dw-1* increased, but there was no significant difference (Figure 6), indicating that ABA may not play a major role in forming the *dw-1* dwarf mutant. By contrast, the IAA and GA contents in *dw-1* were significantly lower (Figure 6), 78.3% and 70.4% of the WT, respectively. Therefore, the decrease in IAA and GA content may be the reason for dwarfing in *dw-1*.

## 3. Discussion

### 3.1. Physiological Changes in Dwarfism of dw-1

The POD enzyme can degrade IAA via oxidation, and its activity directly affects the metabolism and distribution of IAA in plants [30]. High POD enzyme activity can promote the oxidative decomposition process of endogenous IAA, such that when the IAA content decreases, this decrease will inhibit the growth or division of plant cells, resulting in the dwarfing phenotype [31]. In this study, mutant *dw-1* significantly increased POD enzyme activity, so we speculated that the increase in POD enzyme activity might be related to dwarfing in *dw-1*. Relatedly, it has been found that POD accumulation in Zucchini (*Cucurbita pepo*) is negatively correlated with lignin levels [32]. In this study, increased POD activity may have been the main reason for decreased lignin content in mutant *dw-1*. In summary, increased POD activity may be a partial reason for decreased IAA and the height of the *dw-1* plant.

In addition, chlorophyll plays an important role in leaf photosynthesis. The ratio of chlorophyll a to b (Chl *a*/*b*) is an important indicator of plant shade tolerance, and plants with strong shade tolerance have lower Chl *a*/*b* values [33]. The decreased ratio of Chl a to Chl b in the *dw-1* mutant suggests that it may have stronger shade tolerance. Overall, *dw-1* provides valuable material for reducing pruning requirements and lowering management costs.

### 3.2. GA Biosynthesis Is Involved in dw-1 Dwarfing

GA can promote cell division, increase the number of cells, and promote cell elongation, affecting plant morphology and height [34]. There are seven key enzyme genes in the biosynthesis and degradation of GA: *CPS* (*ent copper diphosphate synthase*), *KS* (*ent kaurene synthase*), *KO* (*ent kaurene oxidase*), *KAO* (*ent kaurene acid oxidase*), *GA3ox* (*GA3-oxidase*), *GA20ox* (*GA20-oxidase*), and *GA2ox* (*GA2-oxidase*) [35]. *CPS*, *KS*, and *KO* function in the earliest stage of GA biosynthesis; the *GA1* [36], *GA2* [37] and *GA3* [38] genes encoding them in *Arabidopsis* are single genes, and mutations in these lead to severe dwarfing in plants. Research on rice also shows that the mutations in the *OsCPS1* [39], *OsKS1* [40] and *OsKO2* [41] genes will also decrease plant height and gibberellin content. In this study, one CPS protein and one KO protein were upregulated in *dw-1* leaves, suggesting that the CPS and KO proteins are not the reasons for decreased GA content in *dw-1* leaves. Studies of rice [42,43] and *Arabidopsis* [44,45] show that the enzymes in the late stage of GA biosynthesis (GA20ox and GA3ox) are all encoded by multiple genes, and gene mutation at these sites leads to the semi-dwarf phenotype of plants. In this study, two GA20ox proteins and one KS protein were downregulated in *dw-1* leaves, consistent with decreased GA content in *dw-1* leaves. GA2ox is a key enzyme that decomposes and inactivates GA and its precursors. Overexpressing mutant plants with the *GA2ox* gene, such as rice [44] and *Arabidopsis* [46], have shown a dwarfing phenotype and decreased GA content. In this study, three GA2ox proteins were upregulated in *dw-1* leaves, consistent with decreased GA content in these leaves.

### 3.3. IAA Biosynthesis Is Involved in Dwarfing of dw-1

IAA was the first plant hormone discovered. It plays an important role in plant growth and development, regulating many processes, such as cell division, cell growth, and differentiation [47]. IAA biosynthesis is mainly divided into two pathways, the Trp (tryptophan)-dependent pathway and the Trp-independent pathway, of which the Trp-dependent pathway is the most commonly studied [48]. The indole-3-pyruvic acid pathway (IPA) is the main branch of the Trp-dependent pathway in the IAA biosynthesis of plants, in which TAA and YUC are the key enzymes [49]. In a study of *Arabidopsis taa-* and *yuc*-deficient mutants, the functional deficiencies of *TAA1* and *YUCCA* genes hindered plant growth and development, while the overexpression of IAA-biosynthesis-related genes led to IAA production and accumulation, promoting plant growth [50,51,52]. In this study, two TAA1 proteins and two YUCCA proteins were downregulated in *dw-1* leaves, consistent with decreased IAA content in these leaves.

Plant hormones regulate plant growth and development through synergistic effects [53]. GA signaling primarily relies on the DELLA protein pathway, where DELLA proteins directly inhibit the transcriptional activity of PIF (PHYTOCHROME INTERACTING FACTOR) proteins, promoting IAA biosynthesis [54]. Additionally, during cell proliferation and tissue differentiation, DELLA proteins negatively regulate IAA polar transport through the multi-step inhibition of *PIN* transcription, a crucial component in the IAA signaling pathway [55]. In this study, the GA and IAA contents of *dw-1* significantly decreased. However, whether there is a synergistic effect between GA and IAA that jointly leads to dwarfism in *dw-1* requires further investigation.

### 3.4. Changes in Lignin Synthesis Are Important Factors in Dwarfing

**Figure 7:**
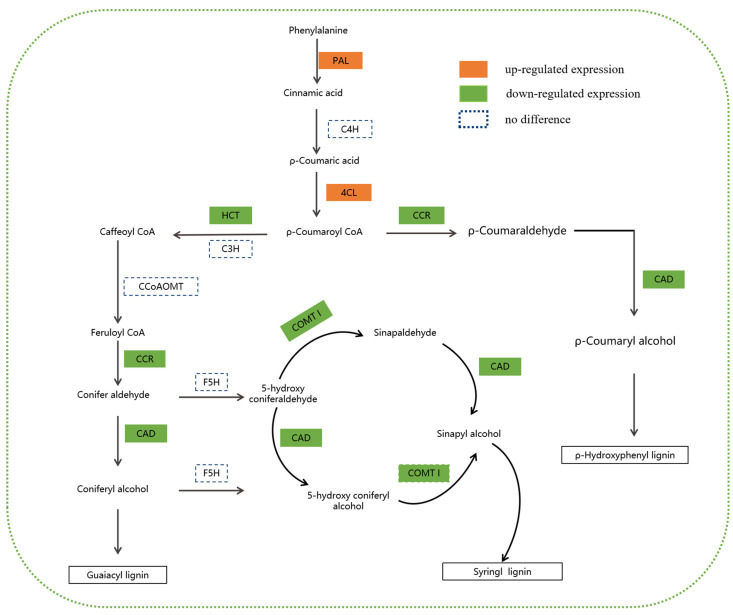
Up- and downregulation of differentially expressed proteins in lignin biosynthesis pathway in *dw-1*. PAL, phenylalanine ammonia-lyase; 4CL, 4-coumaroyl-CoA ligase; HCT, hydroxycinnamoyl CoA: shikimate hydroxycinnamoyl transferase; CCR, cinnamoyl-CoA reductase; CAD, cinnamyl alcohol dehydrogenase; COMT I, caffeic acid O-methyltransferase of class I.

Lignin is an important product synthesized in the phenylpropanoid metabolism pathway of plants, and its synthesis pathway mainly includes the phenylpropanoid metabolism common pathway and the lignin synthesis specific pathway [56]. In the most common pathway of phenylpropane metabolism, PAL and 4CL are rate-limiting enzymes, regulating lignin synthesis. In *Arabidopsis*, the *pal1* and *pal2* single mutants showed no significant phenotypic changes, while *pal1/pal2* dual gene mutant plants exhibited high sterility and significantly reduced lignin accumulation in [57]. *PAL1* gene overexpression and inhibition in *Lotus japonicus* (*Lotus corniculatus*) can lead to changes in PAL activity and lignin content in the roots and nodules of transgenic plants [58]. The *4CL* function of different plants is not the same; however, transgenic plants that inhibit *4CL* activity have shown a decreasing trend in lignin content [59]. Inhibiting the expression of the tobacco (*Nicotiana tabacum*) *4CL* gene can decrease lignin content and the appearance of the dwarfing phenotype [60]. Inhibiting the expression of the *4CL* gene in *Arabidopsis* can decrease lignin content, but *Arabidopsis* will grow normally [61]. In this study, one PAL protein and one 4CL protein were upregulated in *dw-1* leaves, suggesting that PAL and 4CL do not decrease lignin content in *dw-1* leaves (Figure 7).

In the specific lignin synthesis pathway, CCR, HCT, CAD, and COMT are rate-limiting enzymes that participate in lignin biosynthesis [62]. Studies on tobacco [63] and *Arabidopsis* [64] have shown that plants with severely decreased CCR enzyme activity express a dwarfing phenotype, and their lignin content decreases significantly. Research results for *Arabidopsis* [65] and alfalfa (*Medicago sativa*) [66] indicate that plants with silenced or downregulated *HCT* gene expression exhibit decreased plant height, significantly reduced lignin content, delayed growth, and reduced biomass. CAD is the last key enzyme involved in the specific lignin synthesis pathway. The *CAD* gene mutant in *Maize* [67], *Arabidopsis* [68], and *Sorghum bicolor* [69] causes a significant decrease in total lignin content, changes in lignin composition, and changes in normal growth performance. The growth and development of *COMT* gene mutants in *Arabidopsis* [70], *Maize* [71] and *Sorghum bicolor* [72] are normal but accompanied by a decrease in the lignin content, S-lignin content, and S/G lignin ratio. In this study, two CCR proteins, one HCT protein, one CAD protein, and one COMT protein were downregulated in *dw-1* leaves, consistent with the decreased lignin content in these leaves (Figure 7).

There are many ways to regulate gene expression, such as transcription, post-transcription regulation, translation, post-translation regulation, and histone modification [27]. However, transcriptomics studies the expression of genes at the transcriptional level, while proteomics studies the expression of genes at the protein level [28]. Therefore, this study has limitations and deficiencies in exploring dwarfism in *dw-1* through transcriptomics and proteomics alone. Further analysis of *dw-1*’s dwarfing mechanism through other research methods is needed to supplement the current deficiencies in transcriptome and proteomic studies. In future research, our focus will be on achieving gene functional validation by overexpressing or knocking out key genes (*GA2ox*, *GA3ox*, *GA20ox*, *TAA1*, and *YUCCA*) identified through screening, as well as screening key transcription factors through protein interactions.

## 4. Materials and Methods

### 4.1. Plant Material

The test materials were the blue fescue dwarf mutant *dw-1* and its wild-type, ‘Festina’, both of which were self-bred in our laboratory. *dw-1* was obtained via induced mutation with ^60^Co-γ radiation using “Festina”. After two years of field experiments, the dwarfing characteristics of *dw-1* remained stable, so it was used as a test material for this study.

This study began in March 2022 and was conducted at the teaching experimental base of Jiangsu Agri-Animal Husbandry Vocational College in Taizhou, Jiangsu Province, China. To ensure the accuracy of the experiment, WT and *dw-1* plants with good growth and consistent sizes were selected. Before conducting the experiment, we cut off some of the roots and removed any dead leaves. The transplanted seedlings were planted in square plastic pots (length, width, and height of 10 cm) filled with a mixture of vermiculite and peat soil (1:1, *v*/*v*). The WT and the *dw-1* mutant were each planted in 18 pots for 30 days in a greenhouse. The average temperature was 25/20 °C (day/night), relative humidity was 70%, and photosynthetically active radiation was maintained at 800 μmol m^−2^ s^−1^. In management terms, they were watered and fertilized according to conventional methods. The experiment consisted of 6 replicates, each containing 6 pots, totaling 36 pots. After 60 days of stable plant material growth (Figure 1), we collected fresh and mature leaves, immediately froze them in liquid nitrogen for 5 min, and then stored them in a -80-degree ultra-low temperature refrigerator for future use.

### 4.2. Phenotypic Characterization

After taking a tillering bud and culturing it for 30 days, the plant height and leaf length were measured using a vernier caliper. Afterward, the tillers from each plant were counted. The plants were incubated at 80 °C for 15 min and dried at 65 °C for 72 h to measure the shoot and root dry weight. Each phenotypic indicator had 6 biological replicates, with 6 plants measured for each biological replicate and 3 technical replicates measured for each biological replicate.

### 4.3. Transcriptome Analysis

Fresh leaf samples of blue fescue were sent to Frasergen Bioinformatics Co., Ltd. for transcriptome analysis. For transcriptome sequencing, three biological replicates were designed for each sample, and each biological replicate was further divided into three technical replicates. The total RNA of *dw-1* and the WT was isolated and purified using the RNAprep Pure Plant Kit (Tiangen Biotech, Beijing, China), according to the manufacturer’s instructions. The RNA content and purity of each sample were quantified using NanoDrop 2000 (NanoDrop, Wilmington, DE, USA). RNA integrity (RIN > 7.0) was determined using a Bioanalyzer 2100 (Agilent, Santa Clara, CA, USA). A trustee-stranded mRNA sample prep kit (Illumina, San Diego, CA, USA) was used for cDNA library construction. The library was sequenced on an Illumina HiSeqTM 2000 platform (Illumina, San Diego, CA, USA), according to the manufacturer’s instructions. The sequenced data (raw reads) were filtered by removing adaptor sequences, empty reads, reads with more than 5% unknown nucleotides, low-quality sequences (base quality ≤ 20), or sequences with >10% Ns. Clean reads were obtained by filtering raw reads using SOAPnuke software (v2.1.0) [73].

All the downstream analyses were based on high-quality clean reads. Blast2GO [74] was used to annotate the gene functions of unigenes in databases (NR, NT, KOG, and Swiss-Prot). edgeR software (v2.0) [75] was used to identify DEGs between the *dw-1* and WT samples. A corrected *p*-value (padj) of <0.05 and |log2FoldChange| ≥ 1 were the thresholds for significantly differential expression. Gene ontology (GO) enrichment analysis and KEGG enrichment analysis of the DEGs were carried out with reference to Wang’s method [76]. Three groups of parallel measurements were carried out in this experiment.

### 4.4. Protein Extraction and iTRAQ Analysis

Fresh blue fescue leaf samples were sent to Frasergen Bioinformatics Co., Ltd. for proteome analysis. The specific steps of the iTRAQ experimental process are detailed in the attached document (iTRAQ Experimental Process). For proteomic sequencing, three biological replicates were designed for each sample, and each biological replicate was further divided into three technical replicates. Fresh *dw-1* and WT leaf samples were ground into powder with liquid nitrogen, and total protein was extracted with the Plant Total Protein Extraction Kit (Sangon Biotech, Shanghai, China). After the total protein concentration was determined using a Bradford Protein Assay Kit (Thermo Fisher Scientific, Bedford, MA, USA), 100 µg of protein was taken for enzyming and desalting. We dissolved the polypeptide sample with 0.5 mol/L TEAB and marked the iTRAQ according to the instructions of the iTRAQ Reagent-8 plex Multiplex Kit (AB SCIEX, Waltham, MA, USA). The samples were labeled and mixed, and then, the mixed peptides were separated using an Ultimate 3000 HPLC system (Thermo, Waltham, MA, USA) and a Welch C18 column (5 µm, 100 A, 4.6 × 250 mm). Mass spectrometry data were collected for analysis using the triple TOF 5600 plus system (AB SCIEX, Waltham, MA, USA) coupled with the Eksigent nanoLC (AB SCIEX, Waltham, MA, USA).

Screening criteria for DEPs were as follows: When the *p*-value was ≤0.05 and the fold change was ≥1.5 (upregulated expression) or ≤0.67 (downregulated expression), proteins were considered differentially expressed between the two samples. The DEPs identified were functionally annotated based on GO annotations. KEGG pathway analysis of the DEPs was performed based on the Kyoto Encyclopedia of Genes and Genomes (KEGG) database. GO and KEGG enrichment analysis of the DEPs was carried out with reference to Luo’s method [27].

### 4.5. Physiological Index Determination

#### 4.5.1. Determination of Antioxidant Enzyme Activity

Approximately 0.1 g of fresh leaf sample was ground with liquid nitrogen and thoroughly mixed with 1 mL of extraction buffer. After centrifugation at 8000 g for 10 min at 4 °C, the supernatant was taken for enzyme activity detection. The SOD, POD, and CAT activities were measured using visible light spectrophotometry with assay kits (BC0170, BC0090, and BC0200; Solarbio, Beijing, China), according to the manufacturer’s instructions [77]. Each indicator was designed with 6 biological replicates, and each biological replicate was designed with 3 technical replicates.

#### 4.5.2. Determination of Lignin, Cellulose, and Chlorophyll Content

Fresh mature WT and *dw-1* leaves were collected and dried at 80 °C to constant weight. Lignin and cellulose contents were determined using a lignin content detection kit (bc4200, Solarbio, Beijing, China) and a cellulose content detection kit (bc4280, Solarbio, Beijing, China), according to the manufacturer’s instructions [78]. A total of 0.1 g of fresh leaves from the WT and *dw-1* was measured for their chlorophyll content using Lin’s method [28]. Each indicator was designed with 6 biological replicates, and each biological replicate was designed with 3 technical replicates.

#### 4.5.3. Determination of H_2_O_2_ and O_2_^−^ Content

Hydrogen peroxide (H_2_O_2_) content was measured using a H_2_O_2_ content detection kit (Solarbio, BC3590, Beijing, China), taking 0.1 g of the powdered sample and adding the extraction solution. O_2_^−^ content was measured using a O_2_^−^ content detection kit (Solarbio, BC1290, Beijing, China), taking 0.1 g of the powdered sample and adding the extraction solution. The homogenized sample was then centrifuged at 8000 g for 10 min at 4 °C, strictly following the instructions. Each indicator was designed with 6 biological replicates, and each biological replicate was designed with 3 technical replicates.

### 4.6. Determination of Endogenous Hormone Content

Fresh mature WT and *dw-1* leaves were sent to Innovation Biotechnology Co., Ltd., Nanjing, China, to determine their endogenous hormone content, including IAA, ABA, and GA_3_, according to the method of Zhang et al. [79]. The calculation method of hormone content in the sample was as follows: hormone content (ng/g) = detection concentration (ng/mL) × dilution volume (mL)/weighing mass (g). Each indicator was designed with 6 biological replicates, and each biological replicate was designed with 3 technical replicates.

### 4.7. qRT-PCR Analysis for Validation

To validate the transcriptome data, we also collected fresh leaves from each WT and *dw-1* biological replicate again for qRT-PCR analysis [80]. We selected 12 DEGs from the transcriptome for qRT-PCR assays. Total RNA was isolated from the samples using the Fast Pure^®^ Plant RNA Isolation Mini Kit (Vazyme Biotech Co., Ltd., Nanjing, Jiangsu Province, China). RNA was isolated from WT and *dw-1* leaves. For qRT-PCR analysis, the target gene was amplified using the primers listed in Table S8. The control Actin primers were 5′-CTGTACACTGTTCGGACCAT-3′(forward) and 3′ATAGAGATATCGCTTTGTGCAA-5′(reverse). The qRT-PCR conditions were set based on the following parameters: 10 min at 95 °C, 15 s at 95 °C (40 cycles of denaturation), 15 s for annealing at 60 °C, and 20 s for extension at 72 °C. The relative expression level of the target genes and the reference gene was calculated according to the 2^−ΔΔCT^ method [76]. Three biological replicates (with three technical replicates for each biological replicate) were analyzed for each sample.

### 4.8. Data Processing and Analysis

Excel 2010 was used for data organization and preliminary analysis; SPSS 22.0 was used for one-way ANOVA, and Duncan’s multiple comparisons test (*p* < 0.05) was used for analysis.

## 5. Conclusions

In conclusion, our results integrated transcriptomics and proteomics and found changes in genes and proteins related to the *dw-1* dwarf phenotype. DEGs and DEPs were significantly enriched in the diterpene biosynthesis pathway, tryptophan metabolism pathway, and phenylpropanoid biosynthesis pathway, which are mainly involved in the biosynthesis of GA, IAA, and lignin. The downregulation of the GA biosynthesis proteins GA20ox and GA3ox, coupled with the upregulation of the GA degradation protein GA2ox, caused a decline in GA content. The downregulation of IAA biosynthesis proteins TAA1 and YUCCA caused a decrease in IAA content. The decrease in IAA and GA contents may be the main reason for dwarfing in *dw-1*. Their decrease also affects the expression of genes involved in lignin synthesis (*CCR*, *HCT*, *CAD*, and *COMT*), resulting in a decrease in lignin content. The dwarfing phenomenon is directly caused by changes in these genes. These findings provide a foundation for future genetic engineering or breeding strategies aimed at developing dwarf ornamental varieties with reduced management costs.

## Figures and Tables

**Table 1:** Phenotypic characteristics of *dw-1* and the WT.

Morphological Parameters	WT	*dw-1*
Plant height (cm)	27.2 ± 2.1	13.5 ± 1.6 ***
Leaf length (cm)	15.9 ± 1.3	8.8 ± 0.7 *
Tiller number (N/plant)	3.8 ± 1.5	3.6 ± 1.3
Aboveground biomass (g/plant DW)	3.9 ± 0.3	2.8 ± 1.6 *
Underground biomass (g/plant DW)	1.2 ± 0.5	1.1 ± 0.5

Note: * and *** indicate significant differences between the WT and *dw-1* within the same parameters according to Student’s t-test at *p* ≤ 0.05 and *p* ≤ 0.001, respectively (*n* = 6).

**Table 2:** DEPs and their corresponding genes in diterpene biosynthesis.

Gene Name	Protein/Gene Number	Encoding Enzyme	Associated State	Gene LogFC	Protein LogFC
*CPS*	TRINITY_DN31590_c0_g1	ent-copalyl diphosphate synthase [EC:5.5.1.13]	Common up	1.97	1.58
*KS*	TRINITY_DN4711_c0_g2	ent-kaurene synthase [EC:4.2.3.19]	P_down; T_up	2.38	−1.45
*KO*	TRINITY_DN58145_c0_g1	ent-kaurene oxidase [EC:1.14.14.86]	Common up	2.21	1.09
*GA20ox-1*	TRINITY_DN16704_c0_g1	gibberellin-44 dioxygenase [EC:1.14.11.12]	P_down; T_normal	0.33	−1.28
*GA20ox-2*	TRINITY_DN15632_c0_g2	gibberellin-44 dioxygenase [EC:1.14.11.12]	Common down	−9.56	−12.85
*GA3ox*	TRINITY_DN10276_c3_g2	gibberellin 3beta-dioxygenase [EC:1.14.11.15]	P_down; T_normal	0.61	−4.17
*GA2ox-1*	TRINITY_DN101593_c0_g1	gibberellin 2beta-dioxygenase [EC:1.14.11.13]	Common up	4.29	8.66
*GA2ox-2*	TRINITY_DN83662_c0_g1	gibberellin 2beta-dioxygenase [EC:1.14.11.13]	Common up	10.36	11.06
*GA2ox-3*	TRINITY_DN12370_c0_g1	gibberellin 2beta-dioxygenase [EC:1.14.11.13]	P_up; T_normal	0.76	3.84

Note: P, proteome; T, transcriptome. The same applies hereinafter.

**Table 3:** DEPs and their corresponding genes in tryptophan metabolism.

Gene Name	Protein/Gene Number	Encoding Enzyme	Associated State	Gene LogFC	Protein LogFC
*TAA1-1*	TRINITY_DN10102_c0_g1	L-tryptophan-pyruvate aminotransferase [EC:2.6.1.99]	P_down; T_up	2.99	−4.15
*TAA1-2*	TRINITY_DN31940_c0_g1	L-tryptophan-pyruvate aminotransferase [EC:2.6.1.99]	Common down	−5.28	−6.23
*YUCCA-1*	TRINITY_DN24299_c0_g1	indole-3-pyruvate monooxygenase [EC:1.14.13.168]	Common down	−7.20	−8.54
*YUCCA-2*	TRINITY_DN26047_c0_g1	indole-3-pyruvate monooxygenase [EC:1.14.13.168]	P_down; T_normal	0.63	−2.55

**Table 4:** DEPs and their corresponding genes in phenylpropanoid biosynthesis.

Gene Name	Protein/Gene Number	Encoding Enzyme	Associated State	Gene LogFC	Protein LogFC
*PAL*	TRINITY_DN48352_c0_g2	phenylalanine ammonia-lyase [EC:4.3.1.24]	Common up	1.23	1.55
*4CL*	TRINITY_DN14455_c0_g1	4-coumarate--CoA ligase [EC:6.2.1.12]	Common up	2.55	3.47
*CCR-1*	TRINITY_DN4897_c0_g1	cinnamoyl-CoA reductase [EC:1.2.1.44]	Common down	−4.05	−5.09
*CCR-2*	TRINITY_DN4897_c1_g1	cinnamoyl-CoA reductase [EC:1.2.1.44]	P_down; T_normal	−0.65	−2.09
*HCT*	TRINITY_DN18024_c0_g2	shikimate O-hydroxycinnamoyltransferase [EC:2.3.1.133]	Common down	−1.05	−1.79
*CAD*	TRINITY_DN92748_c0_g1	cinnamyl-alcohol dehydrogenase [EC:1.1.1.195]	Common down	−3.06	−4.88
*COMT*	TRINITY_DN9938_c0_g1	caffeic acid 3-O-methyltransferase [EC:2.1.1.68]	Common down	−1.17	−1.58
*peroxidase*	TRINITY_DN5020_c1_g1	peroxidase [EC:1.11.1.7]	Common up	1.12	1.13
*peroxidase*	TRINITY_DN14767_c0_g1	peroxidase [EC:1.11.1.7]	Common up	1.07	1.26
*peroxidase*	TRINITY_DN8357_c0_g1	peroxidase [EC:1.11.1.7]	P_up; T_normal	1.15	1.04

**Table 5:** Physiological differences between *dw-1* and the WT.

Physiological Parameters	WT	*dw-1*
CAT (U·g^−1^·min^−1^ FW)	0.0115 ± 0.0002	0.0130 ± 0.00013
POD (U·g^−1^·min^−1^ FW)	3.7 ± 0.3	5.2 ± 1.1 *
SOD (U·g^−1^·min^−1^ FW)	29 ± 3.3	31 ± 2.3
chlorophyll a content (mg/g DW)	1.385 ± 0.322	1.788 ± 0.351 *
chlorophyll b content (mg/g DW)	0.557 ± 0.022	0.840 ± 0.012 *
Lignin content (%)	23.5 ± 3.1	18.1 ± 2.3 *
Cellulose content (mg/g)	75.2 ± 3.1	55.3 ± 3.1 *
H_2_O_2_ content (μmol/g)	0.56 ± 0.3	0.51 ± 0.28
O_2_^−^ content (μ mol/g)	0.053 ± 0.002	0.047 ± 0.003

Note: * indicates a significant difference between the WT and *dw-1* within the same parameters according to Student’s t-test at *p* ≤ 0.05, *n* = 6.

## Data Availability

All data are presented in this report.

## Supplementary Materials

The following supporting information can be downloaded at: https://www.mdpi.com/article/10.3390/plants13233357/s1, Figure S1: DEGs of diterpene biosynthesis; Figure S2: DEPs of diterpene biosynthesis; Figure S3: DEGs of tryptophan metabolism; Figure S4: DEPs of tryptophan metabolism; Figure S5: DEGs of phenylpropanoid biosynthesis; Figure S6: DEPs of phenylpropanoid biosynthesis; Figure S7: *dw-1* significant difference gene qRT-PCR verification. Ordinate shows the logarithm of differential multiples of the corresponding gene, and the positive and negative values of y axis express the gene up or down, respectively; Table S1: Quality statistics of sequencing data for blue fescue samples; Table S2: Quality statistics of transcript assembly; Table S3: Statistical of gene matching rate; Table S4: Database annotation result statistical table for unigene; Table S5: Summary of protein identification information; Table S6: Functional annotation statistics of totla proteins; Table S7: Statistics of chlorophyll content in *dw-1* and WT; Table S8: Primers for key genes in qRT-PCR.
