# Supplementary material for: RNA-Seq Transcriptomics and iTRAQ Proteomics Analysis Reveal the Dwarfing Mechanism of Blue Fescue (Festuca glauca)
Source: Plants (Basel). 2024 Nov 29;13(23):3357. doi: 10.3390/plants13233357 (PMC11644805; doi:10.3390/plants13233357)
Supplement: Supplementary file 1 [file plants-13-03357-s001.zip › plants-3219204-supplementary.pdf]

plants

MDPI

Table S1 Quality statistics of sequencing data for blue fescue samples

Sample Name	Clean Reads number	Clean Bases number	Clean GC value/%	Clean Q20 value/%	Clean Q30 value/%
WT-1	42849768	12854930400	51.38;51.44	99.20;98.25	97.53;95.40
WT-2	43419918	13025975400	51.99;52.02	99.21;98.02	97.55;94.80
WT-3	25948031	7784409300	52.27;52.30	99.15;98.17	97.40;95.19
DW-1	34431444	10329433200	51.29;51.37	99.19;98.21	97.50;95.31
DW-2	34877617	10463285100	51.60;51.71	99.22;98.15	97.59;95.14
DW-3	32172374	9651712200	52.01;52.13	99.14;98.38	97.37;95.61

Table S2 Quality statistics of transcript assembly

Genes number	Median length	N50 length	Max length	Min length	Average length	Total assembled bases
474231	667	1485	13531	180	985	467156561

Table S3 Statistical of gene matching rate

Sample Name	Total Read pairs	Total mapped reads	Uniq mapped reads	Multiple mapped reads
WT-1	42849768	31511951（73.54%）	5578143（13.02%）	25933808（60.52%）
WT-2	43419918	32639125（75.17%）	5515454（12.70%）	27123671（62.47%）
WT-3	25948031	19579957（75.46%）	3227142（12.44%）	16352815（63.02%）
DW-1	34431444	26002445（75.52%）	4421939（12.84%）	21580506（62.68%）
DW-2	34877617	26319813（75.46%）	4503734（12.91%）	21816079（62.55%）
DW-3	32172374	24389697（75.81%）	4051372（12.59%）	20338325（63.22%）

Table S4 Database annotation result statistical table for unigene

Databases	Number of unigenes	Percentage（%）
NR	247150	52.12
GO	185903	39.20
KO	96037	20.25
KOG	41165	8.68
Swiss-Prot	154211	32.52
Annotated in at least one Database	248029	52.30
Unannotated unigenes	226202	47.70
Total Unigenes	474231	100.00

Table S5 Summary of protein identification information

Run Name	PSMs	Id PSMs	Peptides	Proteins
ALL	396766	103985	22712	3520

Note：ALL: Represents the sum of all sample identifications；PSMs：Total spectrum number；Id PSMs：Number of identification spectra； Peptides/Proteins： The number of identified peptides / proteins.

Table S6 Functional annotation statistics of totla proteins

Identified	GO	KOG	KEGG	Subcell
3520	2796	3341	2653	3520

Table S7 Statistics of chlorophyll content in *dw-1* and WT

Sample	Chlorophyll a	Chlorophyll b	Chlorophyll a/b
dw-1	1.788±0.26*	0.840±0.13*	2.12*
WT	1.385±0.16	0.557±0.09	2.49

Note：* indicate significant difference between WT and *dw-1* within the same Parameters according to Student's t-test at $p \leq 0.05$, N=6 .

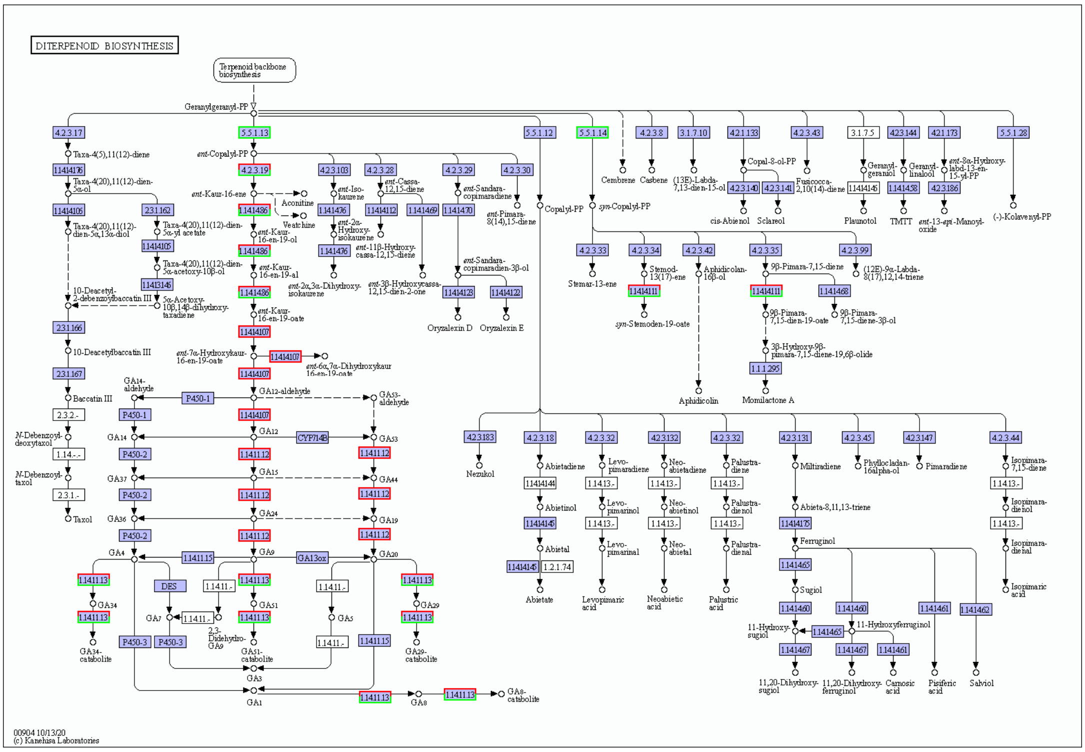

Figure S1 DEGs of diterpene biosynthesis

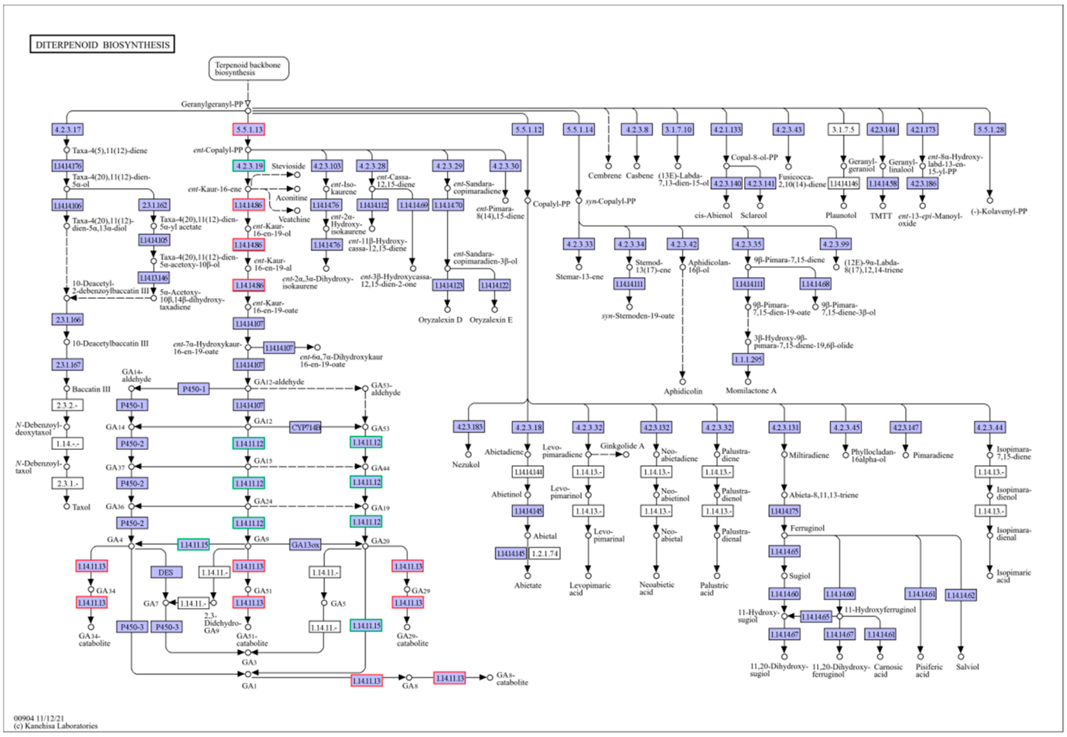

Figure S2 DEPs of diterpene biosynthesis

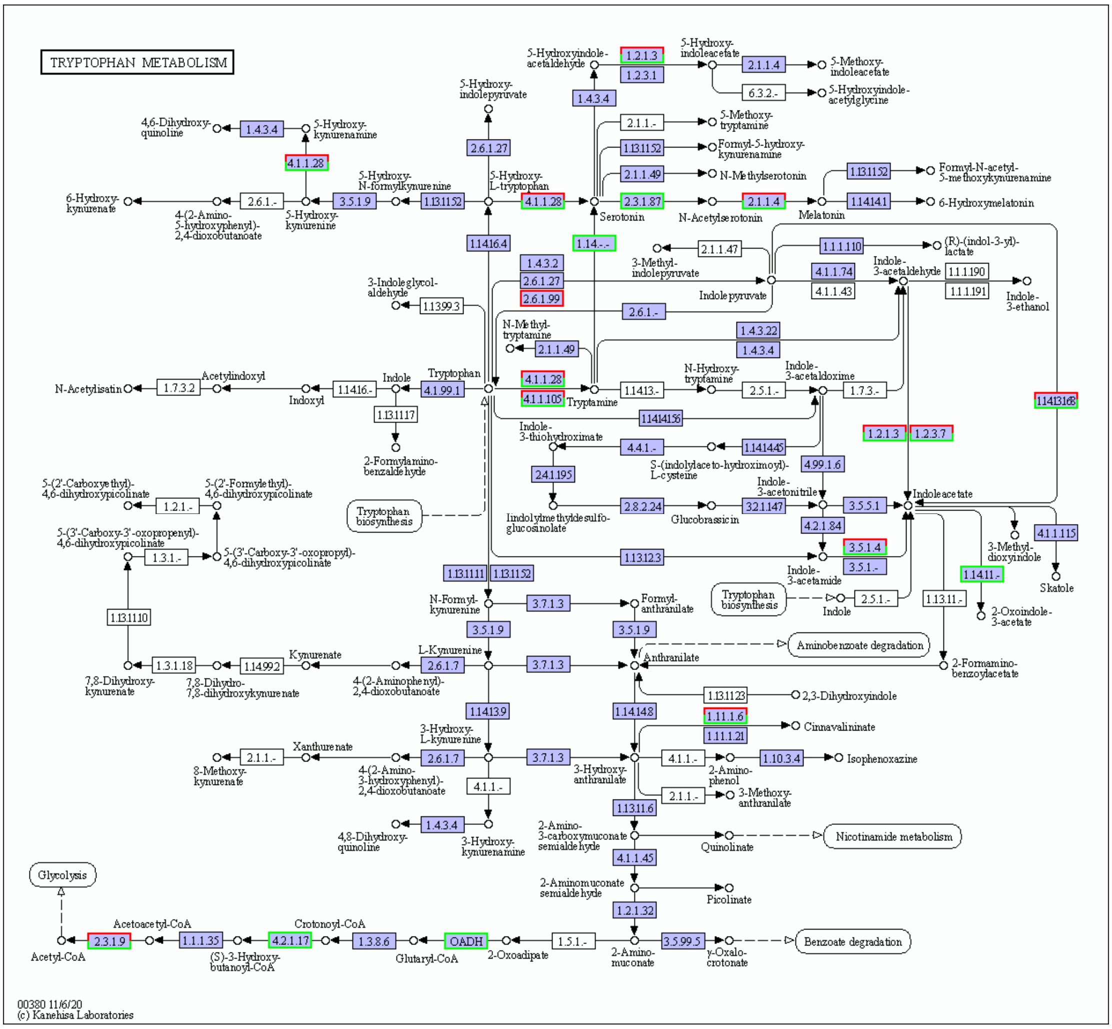

Figure S3 DEGs of tryptophan metabolism

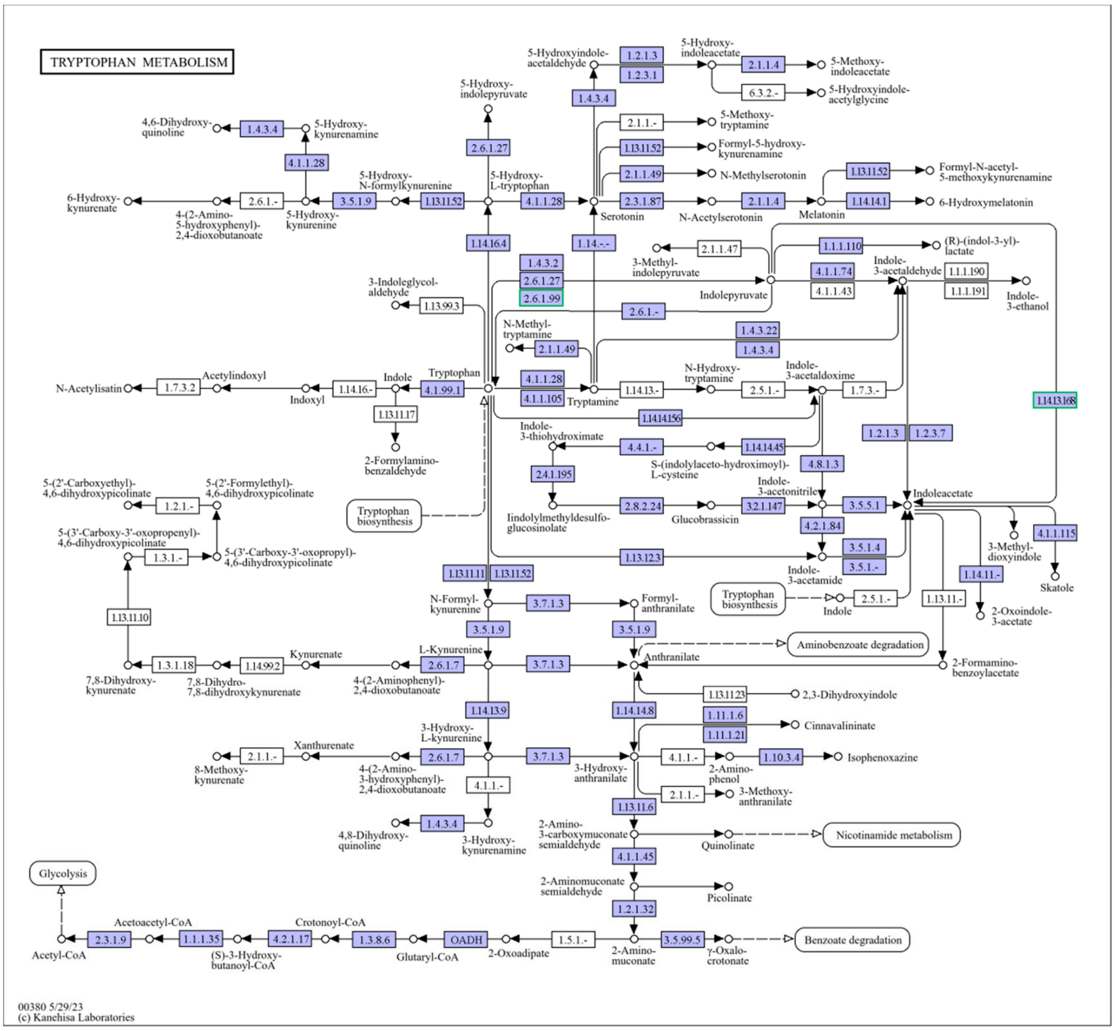

Figure S4 DEPs of tryptophan metabolism

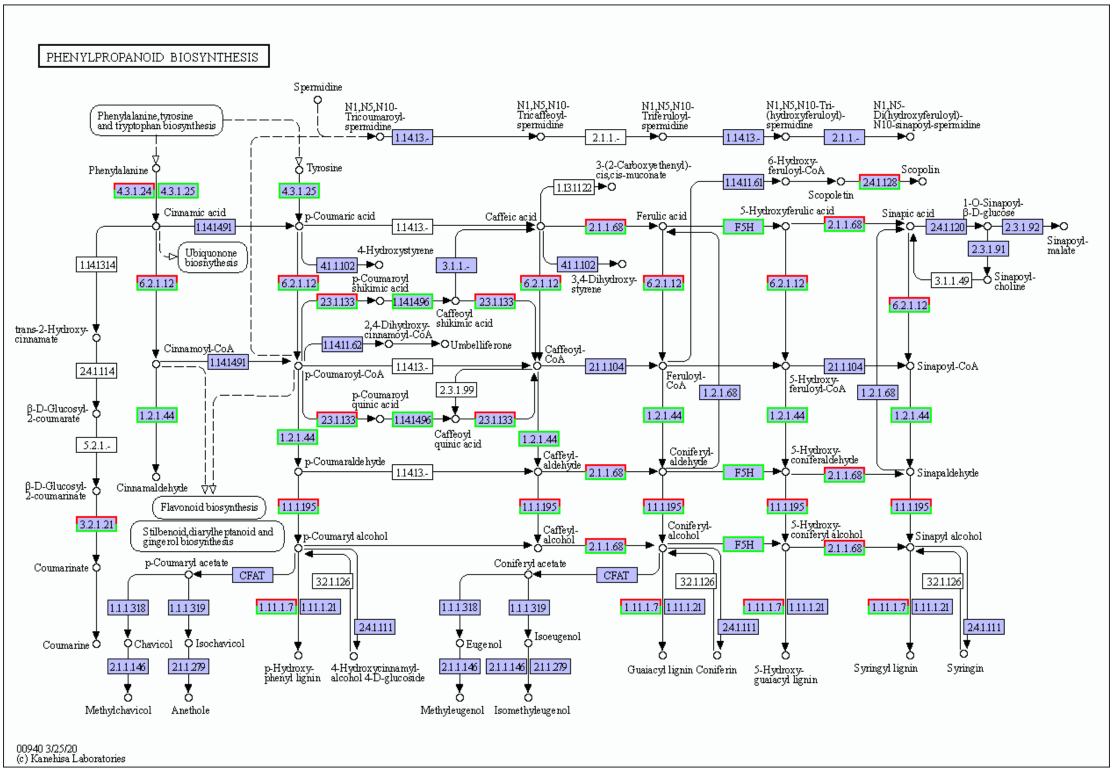

Figure S5 DEGs of phenylpropanoid biosynthesis

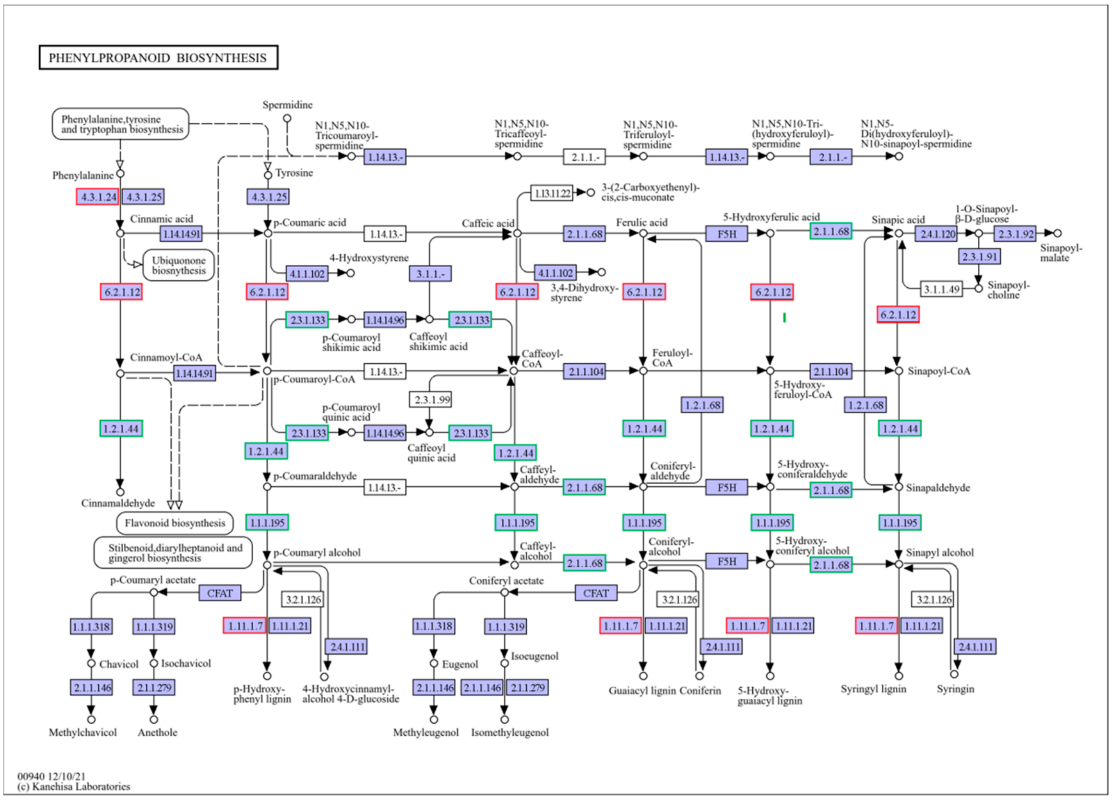

Figure S6 DEPs of phenylpropanoid biosynthesis

Table S8 Primers for key genes in qRT-PCR

Gens ID		Primer
PAL-F	TRINITY_D	TGCTGAGATTGCCATGGCAT
PAL-R	N48352_c0_g 2	TCTTCACATTCTCCTCAAT
COMT-F	TRINITY_D	GGGGTGACCAAGTTCGGGCT
COMT-R	N39130_c0_g 1	GCAGGACGGCATGATGAGT
4CL-F	TRINITY_D	TCGTGGAGAGTGAGATGTCAT
4CL-R	N14455_c0_g 1	CAACAGTTCTACTCTGCCC
CPS-F	TRINITY_D	AGGACCCCCAACCTCCCCGA
CPS-R	N14185_c0_g 1	GCGCCGGCACCGCCAGAGAC
CCR-F	TRINITY_D	GTAGTTGCACAGCTCCTAC
CCR-R	N9118_c0_g1	CAGCACACATGGTACCATCC
HCT-F	TRINITY_D	GCGACCGCGACGGGTGGCGC
HCT-R	N18024_c0_g 2	ATGGTGCCTCTGATCAGGCC
CAD-F	TRINITY_D	TCCACCGACCTTAGCGGCAT
CAD-R	N8703_c0_g1	CTGGAGACGTAGAAGTACAC
KO-F	TRINITY_D	CCGGTTCAAGGTGGCGCTGT
KO-R	N51422_c0_g 1	TAGTTGGACATGCTCGGCCA
YUCCA-F	TRINITY_D	CGTGCACGTGCTCCCGCGCG
YUCCA-R	N26047_c0_g 1	AGCCATTGTGGGACGTTGCT
F5H-F	TRINITY_D	ATGGACCAGCTAACCCACCG
F5H-R	N46228_c0_g 1	GCCGCGGACTCGTCGCGCAC
GA2OX-F	TRINITY_D	ACGGACACGCACGGACTCGT
GA2OX-R	N101593_c0_ g1	AGCTTGGTCTTCTTCCCCGG
GA3OX-F	TRINITY_D	AGATCATTAGCTTGCTCCGA
GA3OX-R	N12370_c0_g 1	GGCTGTGGCTCAGGCGGAGT
Gens		Primer
TIR1-F	TRINITY_D	CGCTCCAGGCACCGCCTCAC
R	N112649_c0 _g1	GGTCATCCTCTCGAAACTCA
GH3-F	TRINITY_D	CTCAAATGTCCGGCGAGACT
R	N12270_c0_ g1	ATCCTCATATGACACGACTG
SAUR-F	TRINITY_D	ATACGCCATGATCACTCCTA
R	N33743_c0_ g1	CCCAAACACAGTTGTACCAA
DELLA-F	TRINITY_D	TTTCTGCTACAGCCCCTTTG
R	N36799_c0_ g1	GAGCTTCTTGCAGCGCTCGG

TF -F	TRINITY_D	AAGATGTTGTAAAAATCATACGA
R	N84400_c0_ g1	CTTCTAAGTTGAGAGATATG
AHP-F	TRINITY_D	TGTTCCTGGACGTGACGGAC
R	N90224_c0_ g1	CTCGATCTTCTCCAGCAGAT
B-ARR F	TRINITY_D	TTCCTGATTCTCAAGTCATTG
R	N85793_c0_ g2	TTCTTGGGGAGGTGGTCCCT
ABF-F	TRINITY_D	CCAGCCTCATCCACGCCCAG
-R	N42808_c0_ g1	ACATGGAGAACATAGCGAGT

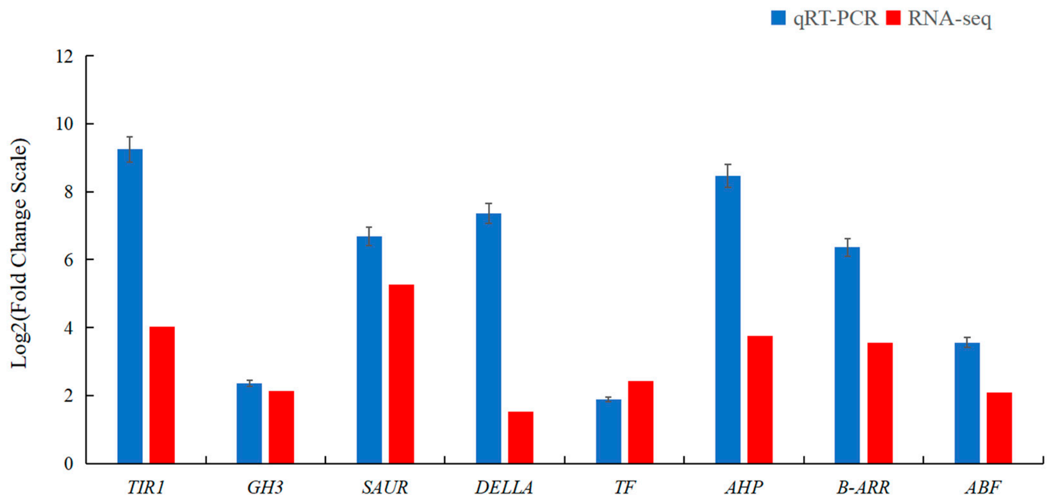

Figure S7. *dw-1* significant difference gene qRT-PCR verification. Ordinate shows the logarithm of differential multiples of the corresponding gene, and the positive and negative values of y axis express the gene up or down, respectively.
